# A comparative study on the development of Chinese and English abilities of Chinese primary school students through two bilingual reading modes: human-AI robot interaction and paper books

**DOI:** 10.3389/fpsyg.2023.1200675

**Published:** 2023-10-04

**Authors:** Yang Feng, Xiya Wang

**Affiliations:** ^1^School of English Studies, Zhejiang International Studies University, Hangzhou, China; ^2^School of Foreign Languages, Guangdong University of Science and Technology, Dongguan, China

**Keywords:** Chinese elementary school students, human-AI educational robot interaction, paper picture books, bilingual reading mode, comparative study

## Abstract

To address the challenges encountered by Chinese primary school students, particularly left-behind and migrant children, who exhibit a preference for animations, video games, and short videos over reading books and struggle with Chinese-English bilingual skills, this study introduces an educational robot AI-assisted method for simultaneous bilingual reading. To assess the effectiveness of this method, a 6-month Chinese-English bilingual extracurricular reading comparative experiment was conducted involving 85 grade 5 students from two classes in a primary school in Hangzhou, China. The AI-assisted class freely read 100 bilingual/English electronic picture books and 200 Chinese electronic classic serial picture books by employing the AI-assisted human-computer interactive electronic reading mode of the “Educational Robot+Audio Electronic Picture Book+Character-play Based Reading.” In contrast, the paper book group read the same content presented in the traditional paper book format, following the “regular independent reading” mode. Post-experimental analyses were conducted employing *t*-tests and MANCOVA and the results revealed that: the primary factors influencing reading effectiveness are the choice of reading materials, reading tools, and reading mode, while reading time does not emerge as the principal influencing factor. Furthermore, students in the AI class demonstrated significant enhancements in bilingual reading motivation, reading amount, reading comprehension, independent learning ability, pronunciation proficiency, and test scores compared to their peers in the paper book class. The AI-assisted reading mode utilizing educational robots garnered positive feedback from teachers, parents, and students. It offers the potential to effectively substitute parental involvement in parent–child reading and English tutoring, while also enabling the simultaneous acquisition of bilingual proficiency in both Chinese and English. This approach proves to be highly effective, cost-efficient, and convenient, particularly for enhancing children’s foreign language abilities. Moreover, it fosters positive reading habits and independent learning skills among primary school students, contributes to the establishment of lofty aspirations, and enhances bilingual performance. Overall, this innovative mode offers an effective means of facilitating children’s acquisition of bilingualism and foreign language skills, as well as promoting reading education.

## 1. Introduction

Elementary school phase is a critical period for linguistic development because it allows children to develop their Chinese-English bilingual ability and good reading habits, as well as their overall academic performance in various subjects, which is crucial to cultivating international talents (Wei, 2019; Xiong and Feng, 2020). The sustainable development of student’s English in middle school often depends on whether students develop an interest and enthusiasm in English learning in primary school (Jiao et al., 2022).

There is currently a substantial educational resource gap between urban and rural areas in China, and the teaching quality of ordinary schools, particularly rural schools, lags well behind that of key schools in large and medium-sized cities. The rural family environment lacks sufficient stimulation for children, with limited access to diverse materials and a scarcity of gaming activities (Wang et al., 2022). Meanwhile, children from blue-collar urban families, rural children who were left behind and raised by grandparents (since their parents moved to the city for work), and migrant children who follow their parents from the countryside to cities for school only have parents with low levels of education and busy work schedules, as well as a lack of awareness and ability of family education, resulting in many elementary school student’s bilingual inability and academic underperformance (Lv et al., 2020). Even in Beijing, the capital of China, most families (90.1%) do not have an English Chinese bilingual family environment (Li G. et al., 2022). Many migrant children either struggle in non-standard informal migrant worker schools or are left in their hometowns, facing difficulties in accessing quality education (Zhang et al., 2021).

Current Chinese-English bilingual education in China confronts numerous challenges, notably stemming from the prevalent employment of traditional paper-based silent picture books devoid of AI robots. This over-reliance on parental and pedagogical Chinese-English proficiency significantly impacts the efficacy of Chinese-English reading and learning. Consequently, the most prevalent scenarios are as follows: (1) These children speak Chinese with a pronounced accent and underperform in Chinese expressiveness, English, and reading comprehension skills, resulting in a lack of independent learning ability, learning initiative, and low academic accomplishment. (2) They procrastinate with their education, despise reading and learning, and have unfavorable reading habits. Every day, 70% of children spend more than 2 h watching anime and playing games on their phones. They rarely read books outside of the course, and the majority of the books they do read are comedies and entertainment. (3) They lack clear goals, motivation to learn, and consideration for their long-term growth (Zhou, 2020; Yang, 2021; Han et al., 2022; Ma and Qie, 2022).

To ameliorate this unfavorable situation, this paper recommends using **the human-computer interactive AI-assisted Chinese-English bilingual simultaneous reading mode** of “shadowing AI audio + group cooperative role play + writing book review/video PK mode” to tackle this problem, and the mode is based on the Cognitive Theory of Multimedia Learning (Mayer, 2009), the Linguistic Critical Period Hypothesis (Cheng, 2017; Abutalebi and Clahsen, 2018; Hartshorne et al., 2018), the Second Language Acquisition Theory of (Krashen, 2018; Patrick, 2019) and the Linguistic Interdependence Theory (Cummins, 2005; Van den Bosch et al., 2020). The **specific measures** involve utilizing an educational robot, designed to engage children’s interest, as the reading tool. Bilingual picture books serve as the reading materials, while AI human-machine interaction serves as the reading mode. The implementation includes carrying out bilingual acquisition through shadowing-reading aloud, using native Chinese language comprehension to facilitate English learning, and improving bilingual pronunciation based on the robot’s scoring corrections. **The purpose of this study** is to offer an economical and convenient method for primary school students, particularly left-behind and migrant children, to simultaneously learn Chinese and English bilingually. Furthermore, the study aims to cultivate a love for reading, instill a habit of independent learning, and foster bilingual proficiency while nurturing ambitious career aspirations and enhancing overall academic performance.

**Figure 1:**
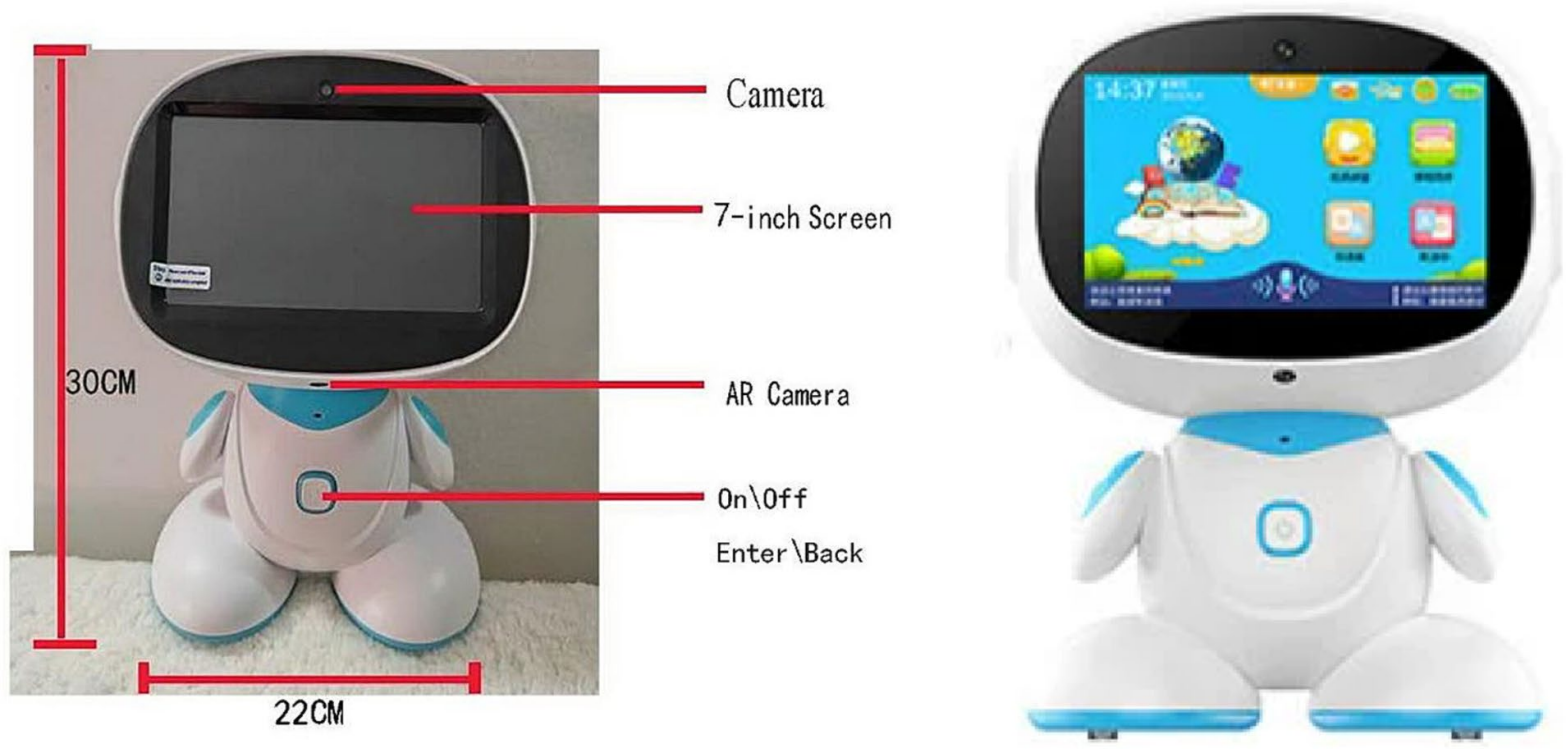
Shape and dimensions of robot.

**Artificial intelligence (AI) educational robots** have recently been developed for AI-assisted learning with a specific focus on reading. It features an engaging robot design, a 7–9 inch touch screen with voice response, dancing and singing capabilities, and a wealth of built-in high-quality reading materials and educational courseware. The robot enables audio reading of bilingual picture books, mutual translation, real-time scoring and correction, as well as the ability to scan augmented reality (AR) image books and textbooks, making it an ideal tool for multilingual instruction and homework assistance (Figure 1). Compared to alternative e-reading software or applications, it offers a heightened level of interactivity.

These human-computer interaction functions enable primary school students in the critical period of linguistic development to enhance their comprehensible input and output by engaging with AI-assisted “shadowing – reading aloud” bilingual electronic picture books provided by educational robots. In addition, the convenient bilingual translation function facilitates foreign language acquisition through the mother tongue. By incorporating audible bilingual electronic picture books, this approach aligns with the cognitive theory of multimedia learning and the design principles of high-efficient learning courseware. Consequently, **the AI-assisted simultaneous Chinese-English bilingual reading mode** receives support from the Cognitive Theory of Multimedia Learning (the integration of multimedia courseware featuring images and sound can yield learning outcomes) (Mayer, 2009), the Linguistic Critical Period Hypothesis (the primary school years represent a pivotal phase for linguistic development) (Hartshorne et al., 2018), the Second Language Acquisition Theory of Krashen (employing intelligible and comprehensive bilingual input–output exercises proves to be an efficient strategy for bilingual acquisition) (Krashen, 2018), and the Linguistic Interdependence Theory (bilingual reading, supplemented by the mother tongue, can effectively aid in foreign language learning) (Cummins, 2005). The robots can limit children’s browsing content and online time to protect their eyesight, as well as serve as a partial substitute for parental accompaniment, which is especially beneficial for busy parents who are unable to tutor their children. This feature distinguishes it from traditional computers, tablets, and cell phones, making it a preferred reading device. The price of educational robots in China varies between ¥1,000 to ¥2,000, depending on the brand and functionality, making it affordable for most families.

To assess the efficacy of the method, a six-month comparative experiment was conducted by the researchers, comparing the effects of bilingual reading using educational robots with AI assistance and conventional paper books. The aim was to confirm the advantages of AI-assisted bilingual reading and contribute to innovative explorations and the accumulation of experience in reforming extracurricular bilingual reading for primary school students in China. This study holds **social significance**.

## 2. Literature review

### 2.1. Support from relevant studies

According to research, bilingual children do not have lower levels of native language competency than monolingual children (Costa and Guasti, 2021), and performance in the native language corresponds with performance in English. Additionally, good literacy practices like parent–child reading aloud together and the number of books in the home are linked favorably to the development of the English language (Wei, 2020; Li H. et al., 2022). First language learning influenced the acquisition of new words in the second language (Xue et al., 2022), and bilingual narrative treatments and vocabulary training were successful in expanding and enhancing children’s bilingual vocabulary (Lipner et al., 2021). ABRA (an acronym for A Balanced Reading Approach for Children Always Designed to Achieve Best Results for All), encompasses an assortment of interactive activities to cultivate foundational literacy proficiencies and writing skills, developed by the Study of Learning and Performance at Concordia University, carried out a comparative study in 339 students. The results showed that the experimental group was better than the control group in key English reading outcomes, such as phonological awareness, early literacy ability, and initial sound fluency (Guo et al., 2023).

These studies apply and refine the Second Language Acquisition Theory of Krashen (2018), and the Linguistic Interdependence Theory (Cummins, 2005), providing support for the AI-assisted bilingual simultaneous reading mode in this study.

### 2.2. The current situation of bilingual reading in elementary schools

The reading of paper picture books is now common in China and under the guidance of teachers, elementary school students reading paper Chinese picture books can improve their Chinese reading comprehension, stimulate the development of text application skills, and have a positive impact on academic advancement (Yao, 2017; Zhang, 2019; Li J., 2021; Li Y., 2021). Meanwhile, primary school students can successfully improve their interest and skill in English reading while also enhancing their English course achievement by reading paper English picture books under the supervision of parents or teachers (Cheng et al., 2018; Zhang, 2018). AR picture books, on the other hand, are a new type of picture book that have emerged recently, and they can significantly stimulate young children’s interest and motivation in reading than paper picture books, the quality of parent–child interaction has a core influence on the effectiveness of young children’s picture book reading (Liu et al., 2019), whereas Yu’s (2018) study found that “discussion-based” parent–child interactive reading mode had better reading effects. Nevertheless, a deficiency in stimulating parenting methods, such as engaging in activities like reading, storytelling, and singing with children, appears to be prevalent in rural and low-income regions of China starting from preschool ages and beyond (Emmers et al., 2021).

The relevant studies mentioned above indicate that encouraging children to read paper/point reading picture books during the critical period of language development can foster their bilingual abilities. However, many primary school students have limited understanding of text reading and show reluctance toward reading paper books. As a result, the effectiveness of their reading often relies on the level of teaching guidance and interaction provided by teachers or parents. Unfortunately, many working families in China may struggle to meet this requirement. Therefore, children from such families require a bilingual acquisition method and teaching aids that do not heavily rely on parental or teacher guidance (Li, 2022; Wang et al., 2022). The **AI-assisted Chinese-English bilingual simultaneous reading mode** in this study provides a cost-effective solution for them.

### 2.3. Computer-assisted bilingual education in elementary schools

Many teachers are now adopting the latest artificial intelligence technologies to change the traditional classroom mode and achieve better learning outcomes than ever before (He, 2016; Jia, 2019; Shu and Gu, 2023). In China, many elementary school teachers use computer-assisted instruction, but their sessions lack human-computer interaction, resulting in inadequate learning (Mei et al., 2018). This demonstrates that the degree of interaction between e-learning courseware and the efficiency of wireless terminal-assisted learning for children is highly reliant (Korat et al., 2014; Zhang, 2021; Ma, 2022).

Electronic textbooks that use the Cognitive Theory of Multimedia Learning to create and present them can help students learn more effectively (Korbach et al., 2017; Liao et al., 2019), and electronic interactive picture books can help children read more effectively than electronic non-interactive picture books and paper picture books (Fang et al., 2019; Deng et al., 2020). Digital games are very popular with all age groups, including when they are used in foreign language education, e.g., Digital Game Based Vocabulary Learning (DGBVL) (Vnucko and Klimova, 2023). The Cognitive Theory of Multimedia Learning (Mayer, 2009) and these studies also showed their support to the **AI-assisted Chinese-English simultaneous bilingual reading mode**. Because its design features and electronic courseware have higher interactivity than tablets, the AI educational robot used in this study is actually a replacement for the tablet learning machine, and its reading material presentation and interaction mode also align more closely with **the 10 principles of the Cognitive Theory of Multimedia Learning** (Hartshorne et al., 2018; Pan and Jiang, 2020).

Presently, research on Internet/computer-assisted English/Chinese education in Chinese primary and secondary schools exhibits the following characteristics: there is a scarcity of studies focusing solely on English or Chinese monolingual teaching, neglecting the aspect of simultaneous acquisition of both languages. Moreover, the majority of these studies are primarily theoretical and lack practical application (Cao, 2015).

Several primary schools in Japan have started exploring the incorporation of AI robots in English teaching. The penguin-shaped small robots utilized lack a touch screen but possess a voice response function, which has garnered popularity among children. Engaging in conversations with these robots offers them more chances to communicate in English, ultimately boosting their enthusiasm for learning a foreign language and alleviating the teacher’s burden (Crace, 2019). However, there is no empirical research on the effectiveness of similar robots in China, and this study aims to address this gap.

## 3. Methodology

### 3.1. Research questions

This study aims to compare the effectiveness of human-computer interaction AI-assisted reading, using “AI educational robot + quality reading materials + bilingual simultaneous acquisition,” with the reading mode of bilingual paper books. It seeks to determine which approach is more effective in developing primary school students’ bilingual ability, reading comprehension, and independent learning skills. The project also aims to cultivate a good habit of reading and independent learning, foster ambitious career aspirations, and comprehensively enhance academic performance. The research questions are as follows:

1. Which reading mode better helps primary school students improve their Chinese-English bilingualism, reading comprehension, and academic accomplishment while also increasing their sustainable passion for bilingual reading?
2. Which reading mode is better in motivating elementary school students to set high goals and develop sustainable and positive reading and independent learning habits?
3. What are the differences in the effects of simultaneous multilingual acquisition and academic development on the two groups of children? What are the key determinants of the outcome? What is the level of student, parent, and teacher acceptance of the reading mode?

### 3.2. Experiment plan

#### 3.2.1. Experiment sampling

Over the course of a winter break + a semester, researchers conducted an after-school reading tutoring experiment in Hangzhou L elementary school with many migrant children (a total of six months). Eighty-five students from two regular fifth-grade classes were randomly assigned to AI and paper book groups, with no significant differences in student numbers or academic achievement. The researchers obtained written consent from the school and all participants’ guardians prior to the experiment. The AI class consists of 43 students, with 22 boys and 21 girls, while the paperback class comprises 42 students, with 22 boys and 20 girls. The gender ratios between the two classes are fairly balanced, and the average age of students was similar (AI class = 11.26 ± 0.36; Paper book class = 11.29 ± 0.31). Additionally, over 60 percent of the students in both classes are children of migrant families from rural areas in different provinces who have relocated to Hangzhou. The remaining students are from local families, but their usual extracurricular reading habits are not satisfactory.

#### 3.2.2. Reading materials

The reading materials were selected based on their substance and were available in both electronic and print media. The AI-assisted class used electronic materials provided by the AI robot, whereas the paper book class used paper reading materials. The Ministry of Education has recommended a collection of outstanding extracurricular reading materials for primary school students (Fan, 2019; Ma, 2021), and from this list, the study has selected the following representative bilingual books:

- One hundred graded bilingual/English picture books, including: 30 *Bilingual Picture Books of World Classic Fairy Tales*, *Where the Wild Things Are* by Maurice Sendak, *Goodnight Moon* by Margaret Wise Brown, *The Very Hungry Caterpillar* by Eric Carle, *The Snowy Day* by Ezra Jack Keats, *Do not Let the Pigeon Drive the Bus* by MoWillems, *Make Way for Ducklings* by Robert McCloskey, *Harold and the Purple Crayon* by Crockett Johnson, *Millions of Cats* by Wanda Gag, *Good Night Gorilla* by Peggy Rathmann, *Three Little Pigs* by Jon Scieszka, *Lilly’s Purple Plastic Purse* by Kevin Henkes, *Owl Moon* by Jane Yolen, *In the Night Kitchen* by Maurice Sendak, *Miss Rumphius* by Barbara Cooney, *Bark, George* by Jules Feiffer, *Corduroy* by Donald Freeman, *No, David* by David Shannon, *Cloudy with a Chance of Meatballs* by Judi and Ron Barrett, etc., over 100,000 English words in total.
- 200 Chinese electronic classic serial picture books and shortened and simplified books for youth, which reflect Chinese and foreign history, great men and heroes, such as *The Biography of Abraham Lincoln*, *The Biography of Lei Feng*, *Romance of the Three Kingdoms*, *100 Stories of Scientists, The Old Man and the Sea, Robinson Crusoe, Hamlet, Yuan Longping, The History of Five Thousand Years, World Natural Geography*, etc., includes over 3 million Chinese characters, as a way to broaden primary school student’s knowledge of history and culture.

#### 3.2.3. AI-assisted class reading mode

The research team provided two educational robots with human-machine interactive AI-assisted reading mode for free to the AI-assisted class to guide students during the lunch break from 12:30–13:30 and after-school services from 15:30–17:30 p.m. every day for about an hour of after-school reading and homework tutoring, as well as record the related work. Students could take turns taking the robot home for use on weekends and vacations and in the evenings. The researchers configured the robot to include a feature that reminds the reader to take a break from the screen after half an hour of continuous use. During this break, the robot automatically switches off for 10 min to protect the children’s eyesight. However, there is no restriction on the number of times the robot can be used in a day.

Fifth-grade elementary school students were chosen as the experimental subjects because many Chinese schools start English classes in third grade, and fifth-grade students have a certain level of Chinese-English bilingual ability, which is conducive to the AI-assisted reading experiment and in line with the Linguistic Critical Period Hypothesis (Hartshorne et al., 2018).

The research group has set up daily reading books and reading plans on the educational robot during the experiment. During the study, students in the AI class used the educational robot and read bilingual picture books in the interactive mode of “AI imitate reading + group collaborative role-play + video PK.” In this mode, students first imitated reading bilingual picture books with the help of the educational robot, reading them once in Chinese and once in English and correcting the bilingual pronunciation based on the robot’s scores; then, several students worked in groups to play roles in picture books and sent the practice videos to the class WeChat group for display, with the goal of improving primary school student’s bilingual speaking and performance skills.

Because fifth graders already have some native Chinese writing skills, they are obliged to express or write down their views on each book (about 300–400 Chinese characters), and then discuss them in small groups to develop teamwork and creativity.

The robot can set and record usage parameters such as time per use, reading content, reading frequency, and score in order to control children’s usage time for effective vision protection and for researchers to analyze the effectiveness and influencing factors of bilingual acquisition experiments.

Members of the research group are responsible for keeping track of journal entries and statistical comparisons, finding and resolving problems, praising active and industrious students, and fostering joint reading, debate, and role-playing. Each group of students can take turns taking the robot home to use it in the evenings, on weekends, and on holidays to improve the efficiency of reading instruction.

#### 3.2.4. Reading mode of paper book class

Simultaneously, the paper book classes were given one set of identical paper books as the AI-assisted class, and daily independent after-school reading was done for around one hour at the same time. However, no robot was provided, and no human-machine interaction reading or assignment tutoring was possible, and the paper books may be taken home by turns for reading.

#### 3.2.5. Test materials and evaluation methods

Questionnaires and interviews on after-school robot tutoring would be conducted both before and after the experiment, as well as an analysis of the experimental subjects’ homework, reading, talent development, learning attitudes, and ideal beliefs, a comparison of final exam scores, and statistical analysis to derive AI robot tutoring and answer the research questions.

##### 3.2.5.1. Bilingual proficiency test

For the pre- and post-experimental tests, researchers created two sets of Bilingual Speaking Proficiency Test Papers for Primary School Students of similar complexity and question style. The test papers were created with reference to The Language Proficiency Test for Children (Liu, 2016), The Grade Standard for Putonghua Proficiency Test (National Language Commission, 1997), and The Cambridge Test Questions for Children’s English Speaking (ESOL Examination Department of Cambridge University, 2014). Each test paper contains 30 questions with a maximum score of 100 points that can be completed in 30 min to examine changes in bilingual competence before and after the experiment. The test paper includes activities such as reading aloud and mutual translation of bilingual words and sentences, talking about visuals, and standard pronunciation. The standard level of Chinese Mandarin and English pronunciation is divided into four levels: “poor,” “average,” “relatively good” and “good.” Cronbach’s Alpha = 0.804>0.7, The reliability value of this test paper is valid (Table 1).

**Table 1:** The reliability value of the test paper.

Number of items	Cronbach’s Alpha
30	0.804

Table 2 displays the results of the Kaiser-Meyer-Olkin (KMO) test, revealing a value of 0.866, which exceeds the acceptable threshold of 0.6, and the significance (Sig) value is 0.000, which is less than the standard alpha of 0.05. Thus, the test paper demonstrates satisfactory validity.

**Table 2:** KMO and Bartlett’s test results for test paper.

Kaiser-Meyer-Olkin measure of sampling adequacy.	0.866
Sig.	0.000

##### 3.2.5.2. Reading comprehension ability

One Chinese and one English reading material were chosen at random and graded on the participant’s general retelling of the primary content as well as events, places, and individuals after reading, with each component receiving 25 points for a total of 100 points.

##### 3.2.5.3. Comparison of academic performance

The evaluation was based on the experimental participants’ final examination scores in school before and after the experiment, with a total score of 100.

##### 3.2.5.4. After-school reading survey

To investigate the robot’s tutoring on homework, talent development, reading, independent learning awareness, and ideal beliefs before and after the experiment, researchers used the Questionnaire on Extracurricular Reading for Elementary School Students (27 questions in 5 dimensions) and the Interview Outline on Extracurricular Reading for Elementary School Students (5 topics).

The questionnaire employed a five-point Likert scale, where the response options ranged from 1 (indicating strongly disagree/dislike) to 5 (indicating strongly agree/dislike). The questionnaire’s Cronbach’s Alpha coefficient was calculated to be 0.823, exceeding the acceptable threshold of 0.7 (Table 3). This indicates that the questionnaire demonstrates strong reliability and validity. The survey and test scoring were conducted by a team of researchers comprising two associate professors/PhDs in English education and two undergraduate trainee teachers specializing in the same subject from Chinese universities. The team followed standardized scoring criteria and conducted a reliability test for scoring.

**Table 3:** The reliability value of the questionnaire.

Number of items	Cronbach’s Alpha
27	0.823

Table 4 displays the results of the Kaiser-Meyer-Olkin (KMO) test, which shows the questionnaire has a satisfactory validity.

**Table 4:** KMO and Bartlett’s test results for questionnaire.

Kaiser-Meyer-Olkin measure of sampling adequacy.	0.816
Sig.	0.000

Semi-structured interviews in Chinese with students in the AI-assisted class were conducted at the conclusion of the study to better understand the strengths and drawbacks of the AI-assisted reading mode. The student interviewees consisted of twenty students in the AI class who were chosen at random, had parental agreement obtained prior to the interview, and were accompanied by both teachers and parents during the interview. Each respondent was interviewed for about 15–20 min. In the interviews, researchers asked questions like, “Do you think the picture books with the aid of AI helped you improve your Chinese and English language skills?” “Do you have any thoughts on how to improve the reading mode?” In order to conduct further content analysis, the interviews were taped and transcribed.

#### 3.2.6. Statistical analysis

In this paper, SPSS 22.0 was utilized to perform an independent samples *t*-test on the aforementioned tests and findings. This analysis aimed to determine the comparability of pre-test scores between the two groups and identify any significant differences in the post-test scores between the AI-assisted class and the paper-based picture book class. Furthermore, a paired samples *t*-test was conducted using SPSS to evaluate whether a significant difference existed between the pre-test and post-test scores of the AI-assisted group, considering the application of both reading modes. The data also underwent comparison and analysis using analysis of covariance. This paper presents a comprehensive statistical analysis to obtain the experimental results and address the research questions. Additionally, it provides an optimized robot tutoring mode. Finally, regarding the analysis of qualitative data, cell phone recordings were used during the interviews. After the interviews, the expressions of all 20 participants were transcribed to explore their learning.

## 4. Experimental results

In this paper, an independent samples *t*-test is first used to assess whether the pre-test scores of the two groups are comparable, and whether there is a significant difference between the post-test scores of the AI-assisted group and the paper-based picture book group. In addition, to determine whether there was a significant difference between the pre-test and post-test scores of the AI-assisted group after using the two reading modes, a paired samples *T*-test was performed.

### 4.1. Chinese reading

The pre-test data revealed that before the experiment, the two groups of participants spent about 2.5 h per day watching cartoons and playing video games, and rarely read extracurricular books, with the occasional extracurricular Chinese reading consisting mostly of Chinese cartoon books, averaging about 0.2 h (12–13 min) per day. In six months, students read 89–90 thousand Chinese characters; the average “Chinese reading comprehension” score is 57–58 points, and the “Chinese final exam” score is 81–82 points. As shown in Table 5, the average “Chinese composition” scores were 25–26 points, with no significant distinction was observed among the comparative indicators.

**Table 5:** Comparison of Chinese learning, extracurricular reading habits, and career aspirations and beliefs before and after the experiment.

Items	Pre-test			Post-test		
	AI class *N* = 43 people	Paper book class *N* = 42 people	Sig.	AI class *N* = 43 people	Paper book class *N* = 42 people	Sig.
Daily time spent watching cartoons and playing video games (hours) Percentage of decrease	2.55 ± 0.32	2.53 ± 0.29	0.283	0.91 ± 0.13 64.32%	1.74 ± 0.30 31.23%	0.000^***^0.000^***^
Daily time for extracurricular reading (minutes)	12.6 ± 1.04	13.2 ± 1.26	0.247	111.6 ± 10.62	69 ± 5.58	0.000^***^
Time ratio between Chinese and English reading	1:0	1:0	0.741	1:1	1: 0.2	0.000^***^
Daily amount of extracurricular Chinese reading (thousand Chinese characters)	0.5 ± 0.06	0.5 ± 0.09	0.763	10 ± 1.22	15 ± 1.41	0.000^***^
Total time for extracurricular reading in 6 months (hours)	38.8 ± 2.71	39.6 ± 3.02	0.316	334.8 ± 32.43	207.0 ± 20.61	0.000^***^
Percentage of increase				785.71%	422.73%	0.000^***^
Total amount of extracurricular Chinese reading in 6 months (thousand Chinese characters)	89 ± 7.63	90 ± 6.36	0.571	1800 ± 117.39	2,700 ± 245.71	0.000^***^
Percentage of increase				2013.5%	2900.0%	0.000^***^
Chinese reading materials	Anime Books	Anime Books		Electronic serial picture books	Paper serial picture books	
Chinese reading mode	A	A		B	A	
Score of Chinese reading comprehension (points out of 100)	57.81 ± 5.23	58.33 ± 5.33	0.417	82.45 ± 7.92	71.62 ± 7.03	0.000^***^
Percentage of increase				42.62%	22.78%	0.000^***^
Interest and motivation in Chinese extracurricular reading	Average	Average		Very high	Relatively high	
Standard level of Chinese Putonghua Pronunciation	Relatively good	Relatively good		Good	Relatively good	0.000^***^
Level of upgrade				1	0	
Scores of Chinese in final exam (points out of 100)	82.16 ± 7.13	81.73 ± 8.16	0.283	93.47 ± 9.11	82.64 ± 8.36	0.000^***^
Percentage of increase				13.77%	1.11%	0.000^***^
Scores of Chinese composition (points out of 40)	25.73 ± 2.76	26.41 ± 2.52	0.242	34.26 ± 3.36	26.75 ± 2.76	0.000^***^
Percentage of increase				33.16%	1.29%	0.000^***^
Status of robot tutoring in various subjects				Good	/	
Activity level in Chinese lesson	Average	Average		Good	Average	
Extracurricular reading habits	Poor	Poor		Relatively good	Relatively good	
Professional ideals and beliefs	Unclear	Unclear		Preliminarily formed	Preliminarily formed	
Chinese autonomous study ability	Average	Average		Very Good	Average	

***p < 0.001; A: Regular independent reading; B: AI-assisted + group cooperative role-play reading + writing post-reading book review.

Following the start of the experiment, students in the AI-assisted class were offered the opportunity to take the robot home for AI interactive reading on a rotating basis. Due to the low cost of educational robots, many children asked their parents to buy one for them, and most parents purchased identical educational robots for their children online at their own expense, resulting in nearly every student in the AI-assisted class having one. The amount of time spent on AI-assisted reading climbed dramatically every day, especially on weekends and holidays, and the frequency, length, and amount of reading done through the AI interactive robot increased significantly. The daily time of AI-assisted reading is much more than 1 h. The average daily extracurricular reading time of the AI class during the experiment reached 1.86 h, almost 9 times the average daily reading time of 0.21 h before the experiment. The average reading time over the six-month experimental period was 335 h, the average daily reading amount was 10,000 Chinese characters, and the total reading amount was 1,800,000 Chinese characters, which was 20 times the reading amount in the same time period before the experiment. The AI educational robot’s capabilities in bilingual translation, audible role-play reading, and scoring the children’s reading motivate the children in the AI class to actively engage with the robot for “bilingual role-play reading” and bilingual translation games. This increased interaction has led to a balanced ratio (1, 1) of reading in Chinese and English, with English proficiency showing significant improvement.

The paper book class spent an average of 1.15 h per day on extracurricular reading during the experimental period, resulting in a total reading time of 207 h, which is 4.2 times more than before the experiment; the total reading amount during the experimental period was 2,700 thousand Chinese characters, which is 29 times more than before the experiment. Since the paper bilingual books lacked pronunciation and translation functions, the children in this group could only read in Chinese and were unable to understand English. Furthermore, they did not participate in role-play reading, which is a crucial element of interactive reading. Although the quantity of reading material surpassed that of the AI class, the effectiveness of their reading experience was significantly lower compared to the AI class, where intensive role-play reading was practiced.

The survey revealed that following the commencement of the reading experiment, children in the AI class were enticed by the engaging reading materials, resulting in a 64% decrease in the time they spent on watching anime and playing video games. Similarly, the paper book class experienced a 31% reduction in these activities. Instead, the children allocated all this freed-up time to engage in bilingual reading. This demonstrates that captivating reading materials, coupled with the AI-assisted reading mode, motivate children to devote more time to reading activities at the expense of their engagement with anime and video games. Undoubtedly, the guidance and encouragement provided by teachers and parents play a significant role in fostering this positive shift.

The AI class’s average reading comprehension post-test score was 82 points, and their average Chinese final exam score was 93 points, including 34 points in composition (out of 40 points), which were 42, 13, and 33% higher than their pre-experimental test scores of 57, 82, and 25 points, respectively. Their “enthusiasm in Chinese extracurricular reading,” “activity level in Chinese class,” and “autonomous study ability” both jumped two levels from “average” to “very high,” and their “Standard level of Chinese Putonghua” (scoring criteria referred to *The Grade Standard for Putonghua Proficiency Test*) improved one grade from “relatively good” to “good.”

The average score of the paper book class on the post-test of reading comprehension ability was 71 points, and the average score on the Chinese final exam was 82 points, including a score of 26.7 points on the composition with a full score of 40, which was 22, 1, and 1% higher than the 58, 81, and 26.4 points in the pre-test respectively; the “enthusiasm in Chinese extracurricular reading” changed from “average” to “relatively good,” which had upgraded one level, but their “activity level in Chinese class,” “Chinese Putonghua speech standard level,” and “autonomous study ability” were still “Average” and remained unchanged.

The pre-test revealed that students in both classes lacked good extracurricular reading habits and clear career goals; however, the post-test revealed that after six months, students in the AI and paper book classes increased their average extracurricular Chinese reading time length by 8 and 4 times respectively, and their amount of Chinese reading increased by 20 and 29 times. Furthermore, through reading a large number of Chinese literature about Chinese history and heroic characters, students developed enhanced extracurricular reading habits and subconsciously absorbed the force of the role models in the books. Most children aspire to be doctors who will heal patients and save lives, engineers who will build the motherland, teachers who will educate children for the country, and so on. Despite the fact that these are just preliminaries, it is often these childish illusions that inspire students to study hard in school and achieve the glory of a future life.

Furthermore, students in the AI-assisted class discovered that the AI educational robot can tutor a variety of subjects, including Chinese and English, mathematics, and science homework, and that they can get answers to questions they do not understand as soon as they ask, and that the robot can answer many questions that parents are unaware of.

### 4.2. English reading

During the six-month experiment, children in the experimental group spent an average of 1.86 h per day on extracurricular reading, with a ratio of Chinese to English reading of about 1:1, with 0.93 h of extracurricular English reading per day, and total reading time of 167 h; the average reading amount was 1,000 English words per day, and the total reading amount was 180,000 words.

The experiment group read over an hour in both English and Chinese, yet the amount of reading in English was only a tenth of the amount of reading in Chinese. This is because Chinese is their mother tongue, and reading comprehension in Chinese is faster, whereas English is a foreign language that they have only recently learned, and reading comprehension in English is slower.

Because English is taught in most Chinese elementary schools beginning in third grade, students’ English vocabulary is limited, and their English reading outside of English textbooks is practically non-existent, resulting in poor English competence. Table 6 demonstrates that the two groups scored 23–24, 32–33, and 72–73 points on “English reading comprehension,” “English speaking ability” (the scoring criteria referred to *The Cambridge Test Questions for Children’s English Speaking*), and “English final exam” respectively, with no significant differences.

**Table 6:** Comparison of English learning before and after the experiment.

Items	Pre-test			Post-test		
	AI class *N* = 43 people	Paper book class *N* = 42 people	Sig.	AI class *N* = 43 people	Paper book class *N* = 42 people	Sig.
Daily time for extracurricular reading (minutes)	12.6 ± 1.04	13.2 ± 1.26	0.247	111.6 ± 10.62	69.3 ± 5.58	0.000^***^
Time ratio between Chinese and English reading	1:0	1:0	0.741	1:1	1: 0.2	0.000^***^
Daily amount of extracurricular English reading (thousand English words)	0	0		1	0.2	0.000^***^
Total time for extracurricular reading in 6 months (hours)	0	0		167.4 ± 14.84	41.4 ± 3.75	0.000^***^
Total amount of extracurricular English reading in 6 months (thousand English words)	0	0		180 ± 10.24	36 ± 2.74	0.000^***^
Reading materials (form of English picture books)				Electronic	Paper	
English reading mode				B	A	
Score of English reading comprehension (points out of 100)	23.62 ± 1.97	24.53 ± 2.41		63.67 ± 6.22	27.71 ± 2.83	0.000^***^
Percentage of increase				169.56%	12.96%	0.000^***^
Interest and motivation in English extracurricular reading	Poor	Poor		Relatively good	Poor	
Score of English speaking test (points out of 100)	32.56 ± 2.74	33.12 ± 3.25	0.153	77.08 ± 8.63	33.84 ± 3.45	0.000^***^
The percentage of increase				136.73%	2.17%	0.000^***^
Standard level of English Pronunciation	Poor	Poor		Relatively good	Poor	0.000^***^
Level of upgrade				2	0	
Scores of English in the final exam (points out of 100)	73.25 ± 12.21	72.94 ± 9.86	0.20	89.74 ± 8.33	73.36 ± 6.81	0.000^***^
Percentage of increase			2	22.51%	0.58%	0.000^***^
Activity level in English lesson	Average	Average		Relatively good	Average	
English autonomous study ability	Poor	Poor		Relatively good	Poor	

***p < 0.001; A: Regular independent reading; B: imitate AI reading + group cooperative role-play + video PK.

The post-test scores for “English reading comprehension,” “English speaking,” and “English final exam” in the AI-assisted class were 63, 77, and 89 points respectively, which were 170, 137, and 23% higher than the pre-test. The scores for “Interest and motivation in English extracurricular reading” and “Standard level of English Pronunciation” both changed from “poor” to “relatively good,” an improvement of 2 levels. English classroom activity moved from “average” to “relatively good,” an improvement of one grade. All of the indicators showed a significant improvement. In an interview, students in the AI class said that this type of English reading helped them develop team spirit by allowing them to engage in more “group cooperative role-play” while reading English.

The post-test scores for “English reading comprehension,” “English speaking,” and “English in final exam” for the paper book class were 28, 34, and 73 points respectively, which were 13, 2, and 0.6% higher than the pre-test. “Interest and motivation in English extracurricular reading” and “Standard level of Chinese Putonghua Pronunciation” remain “poor,” and “Activity level in Chinese class” remains “average,” as it was in the pre-test.

This is because, though the paper book class received the paper bilingual/English picture books with the same contents, they could not spell English and understand the picture book text due to their limited English vocabulary. Despite the inclusion of electronic dubbing in picture books, children need to download and play them on their mobile phones to synchronize with specific pages for audio reading. Children consider this process cumbersome, leading to infrequent engagement with English or bilingual picture books. Consequently, the average reading ratio between Chinese and English stands at approximately 1:0.2. Furthermore, constrained by a limited English vocabulary, children only glance casually at the images in English picture books. In the case of bilingual picture books, their focus is primarily on the Chinese text. As a result, while the average English reading time and amount of the paper book class increased from 0 to 41 h and 36 thousand words in 6 months, their daily English reading time and amount remained low at only 0.2 h and 200 words, making it difficult to improve English reading ability through independent reading of paper English or bilingual picture books. Furthermore, the ranking for “autonomous learning ability,” which remains “poor,” has not changed.

When the English words were touched and clicked by students in the AI-assisted class, the robot’s touch screen read aloud and demonstrated the meaning of the Chinese characters, which was very useful for increasing English vocabulary, learning authentic English sentences, and improving English reading comprehension, and student’s daily English reading time and amount were 0.93 h and 1,000 words, respectively. In six months, the English reading time and amount increased from 0 to 167 h and 180 thousand words, respectively, accumulating enough English reading time and amount to improve English reading comprehension and change the English “independent learning ability” from “poor” before the experiment to “relatively good.” The English “autonomous learning ability” rating changed from “poor” before the experiment to “relatively good,” which is an improvement of 2 levels.

The experiment revealed that the paper book class had an average daily reading time of 1.15 h. However, the ratio of Chinese to English reading was unbalanced (1,0.2), with Chinese reading accounting for 0.96 h and English reading for only 0.19 h. This disparity can be attributed to several factors: the excessive presence of unknown English words in bilingual picture books, the reluctance of children to engage in English reading and role-play reading, and their preference for quickly and silently reading the story contents in Chinese. As a result, the reading effect was subpar. Although there were improvements in Chinese composition skills, the development of Chinese-English bilingual language proficiency, particularly in English, was unsatisfactory.

The AI class had an average daily reading time of 1.86 h, which was 1.6 times longer than that of the paper book class. The ratio of Chinese to English reading in the AI class was 1:1. The Chinese reading time in the AI class is 0.93 h, approximately 0.97 times that of the paper book class, while the English reading time was 0.93 h, 4.9 times higher than that of the paper book class. The presence of an educational robot equipped with AI functions for bilingual pronunciation, translation, and reading aloud scoring motivated children to engage in AI-assisted bilingual character reading practice to receive scoring feedback from the robot. Despite a slower Chinese reading speed and lower reading amount compared to the paper book class, the children in the AI class repeatedly read and engaged with the content through “reading along with the robot” and intensive reading, resulting in a highly satisfactory reading effect.

The experiment revealed that reading time alone does not significantly impact the reading effect. It can be predicted that even if students in the paper class increase their reading time, their bilingual language skills will not reach the level of the AI class. The crucial determinants of the reading effect are the reading mode and the quality of reading materials.

### 4.3. Multivariate analysis of covariance

In this paper, Multivariate Analysis of Covariance (MANCOVA) was employed. This analytical approach is utilized to concurrently consider multiple dependent variables and assess the impact of one or more independent variables on these dependent variables, while also accounting for the influence of covariates. In the context of this paper, pre-test data, reading time, and reading amount are employed as covariates, whereas post-test data are adopted as dependent variables to investigate the effects of distinct variables on post-test Chinese and English performances.

Initially, an assessment of Chinese language scores was executed, and Levene’s chi-square test revealed no significant difference in error variance (*p* > 0.05), confirming the normal distribution of samples (refer to Table 7).

**Table 7:** Homogeneity of Variance Levene’s test for the post-test scores of Chinese.

	*F*	df1	df2	Sig.
Posttest Score of Chinese reading comprehension	0.550	1	83	0.460
Posttest scores of Chinese in final exam	2.838	1	83	0.096
Posttest scores of Chinese composition	3.618	1	83	0.081

Subsequently, the covariates encompassed “pre-test Chinese reading comprehension scores, pre-test Chinese final exam scores, pre-test Chinese composition scores, total amount of extracurricular Chinese reading in 6 months, posttest daily amount of extracurricular Chinese reading.” These covariates were juxtaposed with the dependent variables “post-test Chinese final exam scores, post-test Chinese final exam scores, post-test Chinese composition scores” for the purpose of scrutinizing the impacts of the two reading modes. The findings unveiled substantial associations between the reading modes and the posttest Chinese reading comprehension scores (*F* = 75.972, *p* < 0.001), the posttest Chinese final exam grade scores (*F* = 99.552, *p* < 0.001), and the posttest Chinese composition scores (*F* = 105.563, *p* < 0.001). Moreover, it was determined that the other covariates (including the pre-test Chinese reading comprehension scores, pre-test Chinese final exam scores, pre-test Chinese composition scores, total amount of extracurricular Chinese reading in 6 months, posttest daily amount of extracurricular Chinese reading) displayed no statistically significant interrelations (*p* > 0.05). This highlights the influence of the reading modes on variations in students’ academic performance, surpassing the impact of other variables, as depicted in Table 8.

**Table 8:** Results of MANCOVA for the post-test scores of Chinese.

Source	Dependent variable	Type III sum of squares	df	Mean square	*F*	Sig.
Corrected Model	Posttest score of Chinese reading comprehension	3206.154	8	801.538	21.075	0.000^***^
	Posttest scores of Chinese in final exam	7433.615	8	1858.404	346.805	0.000^***^
	Posttest scores of Chinese composition	1173.433	8	293.358	27.501	0.000^***^
Pretest score of Chinese reading comprehension	Posttest Score of Chinese reading comprehension	157.700	1	157.700	0.146	0.125
	Posttest scores of Chinese in final exam	2.962	1	2.962	0.553	0.459
	Posttest scores of Chinese composition	2.340	1	2.340	0.219	0.641
Pretest scores of Chinese in final exam	Posttest Score of Chinese reading comprehension	25.123	1	25.123	0.661	0.419
	Posttest scores of Chinese in final exam	26.593	1	26.593	0.796	0.092
	Posttest scores of Chinese composition	0.052	1	0.052	0.005	0.945
Pretest scores of Chinese composition	Posttest Score of Chinese reading comprehension	9.758	1	9.758	0.257	0.614
	Posttest scores of Chinese in final exam	0.446	1	0.446	0.083	0.774
	Posttest scores of Chinese composition	2.129	1	2.129	0.200	0.656
Posttest total time for extracurricular reading in 6 months	Posttest Score of Chinese reading comprehension	30.048	1	30.048	0.788	0.377
	Posttest scores of Chinese in final exam	0.338	1	0.338	0.062	0.804
	Posttest scores of Chinese composition	5.165	1	5.165	0.481	0.490
Posttest daily time for extracurricular reading	Posttest score of Chinese reading comprehension	8.412	1	8.412	0.216	0.644
	Posttest scores of Chinese in final exam	8.895	1	8.895	1.634	0.205
	Posttest scores of Chinese composition	1.054	1	1.054	0.096	0.758
Total amount of extracurricular Chinese reading in 6 months	Posttest Score of Chinese reading comprehension	46.118	1	46.118	1.202	0.276
	Posttest scores of Chinese in final exam	1.346	1	1.346	0.249	0.619
	Posttest scores of Chinese composition	0.124	1	0.124	0.012	0.913
Posttest daily amount of extracurricular Chinese reading	Posttest score of Chinese reading comprehension	3.374	1	3.374	0.088	0.768
	Posttest scores of Chinese in final exam	6.254	1	6.254	1.158	0.285
	Posttest scores of Chinese composition	46.596	1	46.596	4.507	0.337
Reading modes	Posttest Score of Chinese reading comprehension	2889.360	1	2889.360	75.972	0.000^***^
	Posttest scores of Chinese in final exam	533.463	1	533.463	99.552	0.000^***^
	Posttest scores of Chinese composition	1126.058	1	1126.058	105.563	0.000^***^
Error	Posttest score of Chinese reading comprehension	3042.570	80	38.032		
	Posttest scores of Chinese in final exam	428.691	80	5.359		
	Posttest scores of Chinese composition	853.373	80	10.667		
Total	Posttest score of Chinese reading comprehension	513916.500	85			
	Posttest scores of Chinese in final exam	536453.000	85			
	Posttest scores of Chinese composition	80367.750	85			
Corrected Total	Posttest score of Chinese reading comprehension	6248.724	84			
	Posttest scores of Chinese in final exam	7862.306	84			
	Posttest scores of Chinese composition	2026.806	84			

***p < 0.001.

In addition, the English performance of both classes was also assessed. Similarly, a Levene’s test for homogeneity of variances was conducted and no significant difference was found in the variances between the two groups (*p* > 0.05) (refer to Table 9).

**Table 9:** Homogeneity of Variance Levene’s test for post-test scores of English.

	*F*	df1	df2	Sig.
Posttest score of English reading comprehension	0.195	1	83	0.708
Posttest score of English speaking test	0.013	1	83	0.909
Posttest scores of English in the final exam	0.121	1	83	0.729

MANCOVA was also employed for conducting analyses involving multiple dependent variables, aiming to evaluate the impacts of various factors on posttest English reading comprehension, English speaking test scores, and English final exam scores. The outcomes revealed: the reading mode exhibited a significant correlation with all of posttest English reading comprehension (*F* = 1100.513, *p* < 0.001), posttest English speaking test scores (*F* = 4372.295, *p* < 0.001), and English final exam scores (*F* = 77.158, *p* < 0.001). This signifies that distinct reading modes yielded substantial effects across the facets of post-test English performances. Conversely, the associations between other variables, including pre-test scores, reading time, and reading amount, and dependent variables were found to be statistically insignificant (*p* > 0.05). This implies that the influence of these factors on post-test scores was comparatively less significant compared to that of reading modes (refer to Table 10).

**Table 10:** Results of MANCOVA for the post-test scores of English.

Source	Dependent variable	Type III sum of squares	df	Mean square	*F*	Sig.
Corrected Model	Posttest score of English reading comprehension	27641.776	8	6910.444	288.495	0.000^***^
	Posttest score of English speaking test	39101.598	8	9775.400	1139.975	0.000^***^
	Posttest scores of English in the final exam	4880.574	8	1220.144	20.560	0.000^***^
Pretest score of English reading comprehension	Posttest score of English reading comprehension	26.710	1	26.710	1.115	0.294
	Posttest score of English speaking test	1.525	1	1.525	0.178	0.674
	Posttest scores of English in the final exam	5.326	1	5.326	0.090	0.765
Pretest score of English speaking test	Posttest score of English reading comprehension	72.339	1	72.339	2.020	0.186
	Posttest score of English speaking test	22.531	1	22.531	2.627	0.109
	Posttest scores of English in the final exam	14.235	1	14.235	0.240	0.626
Pretest scores of English in the final exam	Posttest score of English reading comprehension	33.247	1	33.247	1.388	0.242
	Posttest score of English speaking test	112.506	1	112.506	1.120	0.281
	Posttest scores of English in the final exam	4.699	1	4.699	0.079	0.779
Posttest daily amount of extracurricular English reading	Posttest score of English reading comprehension	18.070	1	18.070	0.745	0.391
	Posttest score of English speaking test	1.314	1	1.314	0.154	0.695
	Posttest scores of English in the final exam	6.239	1	6.239	0.104	0.748
Total time for extracurricular reading in 6 months	Posttest score of English reading comprehension	5.078	1	5.078	0.209	0.649
	Posttest score of English speaking test	20.804	1	20.804	0.446	0.122
	Posttest scores of English in the final exam	46.639	1	46.639	0.775	0.381
Total amount of extracurricular English reading in 6 months	Posttest score of English reading comprehension	7.459	1	7.459	0.319	0.574
	Posttest score of English speaking test	1.892	1	1.892	0.216	0.644
	Posttest scores of English in the final exam	33.941	1	33.941	0.573	0.451
Daily amount of extracurricular English reading after experiment	Posttest score of English reading comprehension	71.413	1	71.413	0.059	0.094
	Posttest score of English speaking test	0.004	1	0.004	0.067	0.984
	Posttest scores of English in the final exam	67.230	1	67.230	1.136	0.290
Reading modes	Posttest score of English reading comprehension	26361.025	1	26361.025	1100.513	0.000^***^
	Posttest score of English speaking test	37492.855	1	37492.855	4372.295	0.000^***^
	Posttest scores of English in the final exam	4578.985	1	4578.985	77.158	0.000^***^
Error	Posttest score of English reading comprehension	1916.272	80	23.953		
	Posttest score of English speaking test	686.008	80	8.575		
	Posttest scores of English in the final exam	4747.620	80	59.345		
Total	Posttest score of English reading comprehension	207903.250	85			
	Posttest score of English speaking test	301220.500	85			
	Posttest scores of English in the final exam	597103.750	85			
Corrected Total	Posttest score of English reading comprehension	29558.047	84			
	Posttest score of English speaking test	39787.606	84			
	Posttest scores of English in the final exam	9628.194	84			

***p < 0.001.

### 4.4. Acceptance of the two reading modes

According to the post-test, 74% of students in the AI-assisted class highly appreciated the human-computer interactive reading mode, 14% showed a preference, and only 11% detest it. Opponents prefer to learn English by reading or reciting the English sentences in the picture book on their own, and they think that cooperative role-playing in a group is too tedious and inefficient. Teachers and parents, on the other hand, unanimously agreed that the AI-assisted class’s human-computer interactive role-play reading mode reduced their teaching and tutoring workload, cultivated children’s good reading habits and cooperative learning team spirit, and effectively promoted children’s bilingual ability development, as shown in Table 11.

**Table 11:** Attitudes of students, parents, and teachers toward the two reading modes.

Survey results	AI class *N* = 43 people		Paper book class *N* = 42 people	
	Number (people)	Percentage (%)	Number (people)	Percentage (%)
Students who highly appreciated the mode and found it to be effective	32	74.42	18	42.86
Students who showed a preference for this reading mode and perceived it to be more effective	6	13.95	13	30.95
Students who expressed moderate satisfaction with the mode and reported average outcomes	5	11.63	11	26.19
Students who expressed a desire to continue using the mode in subsequent periods	41	95.35	31	73.81
Parents who endorse this reading mode (equal to the number of students)	43	100.00	33	78.57
English teachers who support the mode (6 people in total)	6	100.00	4	66.67

Conversely, in the paper book class, 43 and 31% of students expressed a positive liking and preference for the reading mode, considering it effective as well (It should be noted that this pertains to the stage when they are not engaged in AI-assisted robot reading). However, 26% of the students had a less favorable opinion. The percentage of parents and teachers who regarded the reading mode as effective was 78 and 67% in comparison to the AI class.

### 4.5. Analysis of interview results

#### 4.5.1. AI class

In group interviews with a random sample of 20 students in the AI class, 15 of them reported, “Reading it with the AI robots was very fun. When we came across unknown words, especially new English terms, we would touch the robot’s screen and look at the Chinese translation, so that the meaning of the new words could be absorbed, which was useful for understanding the story.” By stating that students use their Chinese knowledge to acquire and understand English while reading, this answer supports the Linguistic Interdependence Theory.

Thirteen students stated, “We can now read picture books independently and at our own pace thanks to the AI robot. We also imitated parts of the English dialogs, which helped us improve our English.” While students in the paper book class could not continue to read when they encounter unfamiliar English words. As a result, they just read the Chinese portion of the book and do very little reading in English.

Seventeen students said: “Reading with robots was more engaging and interactive than reading textbooks or listening to lectures. It did not feel like we were learning. It felt like we were playing games with robots. During the reading interaction, time would fly by. We reached the half-hour reading time limit very soon each time.” This demonstrates that the AI reading mode allows students to naturally interact with and acquire Chinese and English bilingualism while playing the game and interacting with learning robots, and they enjoy it.

Fifteen children expounded on how “AI-assisted e-reading has elevated our reading comprehension and instilled positive reading habits. Simultaneously, our inherent zeal for reading and grasp of traditional paper books have witnessed further enhancement.” This observation underscores that, even as the children derive pleasure and heightened comprehension from the AI-assisted reading mode, their affinity for paper-based reading persists and, in fact, experiences a surge in interest.

Finally, eleven students stated: “Our parents’ English is not good enough to read English picture books for us. We can now rely on the AI robot to read English for us, and we have learned quite a few new (English) words.” When compared to typical paper picture books without audio, many students stated they spent 2–3 h reading over the weekend, implying that AI robot assisted reading can motivate children to read, help them build independent reading habits, and significantly improve their English skills.

#### 4.5.2. Paper-book class

Group interviews were conducted with a randomly selected sample of 20 students from this class. All participants expressed their positive interest in the provided paper bilingual books. They believed that reading the books broadened their perspectives and enhanced their Chinese language skills. However, they did not observe significant improvements in their English language skills. They found the presence of numerous unknown English vocabulary words to be challenging, making it inconvenient to use a dictionary, resulting in their focus primarily on the Chinese content. Moreover, they found the role-play reading of Chinese characters to be challenging, preferring to read Chinese storybooks quickly without engaging in intensive reading. Consequently, while they were exposed to a wide range of content and broadened their horizons, they struggled to retain specific information, resulting in limited improvement in examination results.

## 5. Discussion

In this study, *t*-tests were used to compare and analyze the performance of the AI-assisted class with the paper book class in Chinese and English reading comprehension, Chinese and English final exams, Chinese essay writing and spoken English and other areas before and after the experiment. Both the paper book and AI-assisted classes demonstrated significant improvements in their multilingual and reading abilities, supporting the Linguistic Critical Period Hypothesis. The findings further affirm that reading contributes to the children’s linguistic development and illustrate that the human-AI robot interactive bilingual reading mode can enhance the interest and motivation of Chinese elementary school students in bilingual reading of Chinese and English, while also improving their bilingualism, reading comprehension, independent learning ability, and academic achievement. These results showcase the potential of the “AI-assisted reading” mode to help elementary school pupils establish clear life goals, develop strong reading habits, and learn independently, while also supporting their academic progress and professional aspirations.

By synthesizing the MANCOVA outcomes for both Chinese and English, this paper investigates the influence of various factors on students’ linguistic achievements. Through a comprehensive examination of the findings across both Chinese and English, a crucial discovery has emerged: reading modes exert a substantial impact on students’ performance. This assertion holds true for both Chinese and English, where the reading mode demonstrates a statistically significant influence on students’ reading comprehension, oral test scores, and final exam scores, surpassing the impact of other factors.

The AI-assisted class showed a more significant advancement in bilingual development compared to the paper book class. A pivotal contributing factor to this phenomenon was the efficacious implementation of role-play bilingual reading. Educational robots proficiently evaluated the bilingual reading, increased linguistic interaction between human and AI robots, and motivated children to engage in more bilingual activities and readings. This heightened language input–output practice fostered increased language interaction, thereby enhancing their bilingual proficiency. This practice aligns with the principles of bilingual acquisition theory. Notably, the role-play reading occurred organically among the participants, rather than being orchestrated by the teacher. The AI class readily embraced role-play reading due to the bilingual pronunciation translation and AI scoring capabilities of the educational robot. The allure of human-AI robot interaction and the desire for the robot’s evaluation spurred their engagement. In contrast, paper books lacked these functionalities, causing the children to perceive role-play reading as challenging and unappealing. This distinction underscores the divergent reading effects elicited by distinct reading modes.

The selection of the AI robot as a tool was based on the Cognitive Theory of Multimedia Learning’s dual-channel hypothesis (the robot is video+audio), limited-capacity hypothesis (the AI robot does not show irrelevant web pages while helping children read and can avoid distractions), and active-processing hypothesis (children actively process new knowledge and integrate it with what they already know). The high level of bilingual acquisition and academic achievement demonstrated by children in the AI-assisted class compared to those in the paper book class provides evidence for the validity of this hypothesis. The positive feedback from over 90% of the students, parents, and educators further supports the effectiveness of this approach.

Anticipating the interactive nature of AI robots, the researchers hypothesized prior to the experiment that the AI-assisted class would exhibit longer reading times and greater reading volume. Contrary to the prediction, the total reading time during the experiment in the paper book class was 30% less than that in the AI class, but the amount of reading increased by 50% compared to the AI class. This discrepancy is attributed to the captivating and appealing reading materials used in the experiment, such as paper-based bilingual picture books and comic books. Children in the paper book class demonstrated a keen interest in the materials and read nearly all of the books. However, the conventional “independent reading” mode, which entails reading a book in a short amount of time, did not yield the same results as the AI group’s “AI-assisted + group reading + writing after reading/video PK” mode. As a result, the average score for “reading comprehension” in the post-test of the paper book class was 71, which was 15% lower than the score of 82 in the AI class.

Both the AI-assisted and paper book groups showed a decrease in daily time spent watching cartoons and playing video games as their extracurricular reading time increased. Specifically, the AI-assisted class had an average decrease of 25% or 0.64 h, while the paper book class had an average decrease of 11% or 0.29 h. On the other hand, both groups showed an increase in their active participation levels during the reading sessions, which is a phenomenon that parents and teachers are pleased to see. However, despite the positive outcomes, it is important to note that reading still has not fully replaced video games and animation as sources of entertainment for children.

According to student interviews, their interactions with AI robots are more akin to “play” than “learning,” indicating that the AI interactive reading mode made students willing to spend more time on the natural acquisition of Chinese and English bilingualism through entertainment and play, which is a significant distinction between this reading mode and other existing language learning methods. It is the reason why the total “reading” time of the AI class is 1/3 more than that of the paper book class and the ratio of Chinese to English reading is basically 1:1.

The results suggest that the reading effects achieved by the AI robot-assisted reading mode are considerably superior to those achieved by Japanese primary schools using penguin-shaped robots for instructional purposes, particularly in English language learning (Crace, 2019). The AI-assisted language acquisition and reading education is more effective and popular than other computer-assisted instruction methods (Mei et al., 2018), indicating a more promising new paradigm.

## 6. Conclusion

In the six-month bilingual extracurricular reading experiment, the AI-assisted and paper book classes’ performance was evaluated and the pre-and post-test data revealed:

(1) The AI-assisted class showed a significant improvement in Chinese reading time and amount of reading, which increased by 8 and 20 times respectively, compared to the paper book class whose readings increased by 4 and 29 times, respectively. In addition, the AI-assisted class demonstrated a 43% improvement in reading comprehension ability, while the paper book class improved by 23%. The Chinese final examination and composition scores of the AI-assisted class increased by 14 and 1%, compared to the paper book class’s scores which improved by 33 and 1%. Their standard level of Chinese Putonghua pronunciation, extracurricular reading enthusiasm, and learning activity in English class improved by 2 grades and 1 grade, respectively. Meanwhile, the “Chinese autonomous learning ability” of the AI-assisted class improved by 2 grades, while the paper book class showed no improvement in this area.

The study also has found that the AI-assisted class experienced a marked increase in their English reading time and amount, with an increase from 0 to 167 h and 180,000 words, respectively. In comparison, the paper book class increased their English reading time by 41 h and read 36,000 words. The AI-assisted class also demonstrated an improvement in English final examination and spoken scores, which increased by 23 and 137%, respectively. Their standard level of English pronunciation, extracurricular reading enthusiasm, and English class learning activity also improved by 2 grades and 0 grade respectively, while their “English autonomous learning ability” improved by 1 grade and 0 grade, respectively. Moreover, the AI-assisted class exhibited a decrease in their daily time spent watching cartoons and playing video games by 25 and 11%, respectively. This class also demonstrated the emergence of a career ideal. It is noteworthy that the improvement range for many indicators was substantially greater in the AI-assisted class as compared to the paper book class.

(2) The implementation of this human-AI robots interactive bilingual reading mode for elementary school students in China effectively and simultaneously promoted their Chinese and English bilingual ability, reading comprehension, independent learning ability, active classroom participation, and academic achievement. The mode encouraged students to set high goals and develop good reading habits, which can have a positive impact on their academic growth and performance.
(3) In the AI-assisted class, the human-AI robots interactive bilingual reading mode was popular among students, parents, and teachers, and it is a new way to effectively assist children’s bilingual/multilingual acquisition and reading education. The most influential factors of reading effectiveness are the choice of reading materials, reading tools, and reading mode, while reading time is not the main influencing factor. The AI educational robot can help children with extracurricular reading and academic tutoring in some ways.

Rather than focusing solely on paper picture books or electronic devices, this study compares AI robots with paper picture books, addressing a research gap. It is found that AI robot interactive reading mode can enable children to naturally and simultaneously acquire Chinese and English in play and entertainment, and can effectively cultivate Chinese student’s bilingual ability. This effort provides a novel approach to English learning for children from non-English speaking countries, and it could serve as a mode for English instruction in other non-English speaking countries.

Future research can continue to explore whether this benefit can be maintained over a long period of time, and whether the power provided by AI robots can be internalized into children’s intrinsic motivation. It can also consider increasing the number of students participating in the experiment, selecting more schools of different levels in different regions, and extending the experimental time to better understand the impact on students’ long-term bilingual development.

## Data availability statement

The original contributions presented in the study are included in the article/supplementary material, further inquiries can be directed to the corresponding author.

## Ethics statement

The studies involving humans were approved by the Zhejiang International Studies University, Guangdong University of Science and Technology and Hangzhou Liuxia Primary School, in accordance with the Declaration of Helsinki. The studies were conducted in accordance with the local legislation and institutional requirements. Written informed consent for participation in this study was provided by the participants’ legal guardians/next of kin.

## Author contributions

YF: conceptualization, data curation, formal analysis, funding acquisition, methodology, project administration, and validation. XW: investigation, resources, interview, and reading guidance. YF and XW: writing original draft, reviewing, and editing. All authors have read and agreed to the published version of the manuscript.

## References

[ref1] Abutalebi J. Clahsen H. (2018). Critical periods for language acquisition: new insights with particular reference to bilingualism research. Biling. Lang. Congn. 21, 883–885. doi: 10.1017/S1366728918001025

[ref2] Cao W. (2015). Status quo of computer-assisted ELT in basic education in China. Technology Enhanced Foreign Language Education 4, 41–46. doi: 10.3969/j.issn.1001-5795.2015.04.007

[ref3] Cheng B. (2017). Updating modern education ideas from the perspective of Critical Period Theory. Educ. Theory Pract. 37, 17–20.

[ref4] Cheng Z. Guan Q. Wang D. (2018). An analysis of English picture book reading for primary school students. J. Changchun Inst. Educ. 34, 76–78. doi: 10.3969/j.issn.1671-6531.2018.04.024

[ref5] Costa F. Guasti M. T. (2021). Is bilingual education sustainable. Sustainability 13:13766. doi: 10.3390/su132413766

[ref6] Crace A. (2019). Robots to Help Teach English in Japan [Online]. Available: https://thepienews.com/news/japan-employ-robots-elt/ (Accessed January 10, 2023).

[ref7] Cummins J. (2005). “Teaching for Cross-language Transfer in Dual Language Education: Possibilities and Pitfalls”, in: *TESOL Symposium on dual language education: Teaching and learning two languages in the EFL setting*. (Estambul: Universidad Bogazici Turquía). Available at: https://gustavorubinoernesto.com/wp-content/uploads/2020/06/Teaching-for-Cross-Language-Transfer-in-Dual-Language-Education-Possibilities-and-Pitfalls-Jim-Cummins.pdf

[ref8] Deng Y. Gao H. Qu F. Jiang Y. (2020). A study on the difference between paper picture books and electronic picture books for 5-6 year olds. Fujian Educ. 42, 12–19.

[ref9] Emmers D. Jiang Q. Xue H. Zhang Y. Zhang Y. Zhao Y.. (2021). Early childhood development and parental training interventions in rural China: a systematic review and meta-analysis. BMJ Glob. Health 6:e005578. doi: 10.1136/bmjgh-2021-005578, PMID: 34417271 PMC8381307

[ref10] ESOL Examination Department of Cambridge University. (2014). Cambridge Children’s English full test. Beijing: Foreign Language Teaching and Research Press.

[ref11] Fan Y. (2019). Serialized texts: Speculative reading in mysterious anticipation. Res. Element. School Teach. 13, 19–21.

[ref12] Fang R. Zhang S. Wei X. (2019). Research on the design of electronic picture books and paper picture books based on children’s reading differences. Publish. Sci. 27, 71–77. doi: 10.13363/j.publishingjournal.2019.05.012

[ref13] Guo X. Cheung A. C. K. Abrami P. C. Wade A. (2023). Examining the impact of ABRACADABRA (ABRA), a game-based online literacy program, on primary school students in rural Hunan, China. Educ. Technol. Res. Dev. 71, 1297–1322. doi: 10.1007/s11423-023-10185-5

[ref14] Han F. Jiao F. Yang W. Yang Y.-Z. Deng R. (2022). A study on the current neglect status of urban migrant children aged 6-11 years in three states (cities) of Yunnan Province. Chinese J. Child Health 12, 1376–1379+1384. doi: 10.11852/zgetbjzz2021-1395

[ref15] Hartshorne K. Tenenbaum B. Pinker S. (2018). A critical period for second language acquisition: evidence from 2/3 million English speakers. Cognition 177, 263–277. doi: 10.1016/j.cognition.2018.04.007, PMID: 29729947 PMC6559801

[ref16] He J. (2016). The application of tablet learning terminal in elementary school English teaching activities: A case study of the lesson for Healthy Life. Educ. Inform. Technol. 12, 63–69. doi: 10.3969/j.issn.1671-3176.2016.12.019

[ref17] Jia J. (2019). Exploring the application of tablet computers in English classroom teaching in primary and secondary schools--an analysis based on nine videos of English courses in primary and secondary schools in six provinces and cities. Mod. Educ. Technol. 29, 74–79. doi: 10.3969/j.issn.1009-8097.2019.11.011

[ref18] Jiao S. Jin H. You Z. Wang J. (2022). Motivation and its effect on language achievement: sustainable development of Chinese middle school students’ second language learning. Sustainability 14:9918. doi: 10.3390/su14169918

[ref19] Korat O. Levin I. Atishkin S. Turgeman M. (2014). E-book as facilitator of vocabulary acquisition: support of adults, dynamic dictionary and static dictionary. Read. Writ. 27, 613–629. doi: 10.1007/s11145-013-9474-z

[ref20] Korbach A. Brünken R. Park B. (2017). Measurement of cognitive load in multimedia learning: a comparison of different objective measures. Instr. Sci. 45, 515–536. doi: 10.1007/s11251-017-9413-5

[ref21] Krashen S. (2018). The conduit hypothesis: how reading leads to academic language competence. Language Magazine 4, 127–141.

[ref22] Li J. (2021). A Study on the Use of Chinese Comic Strips in Language Reading Teaching in Lower Middle School. [Master’s thesis]. Kashi: Kashi University.

[ref23] Li Y. (2021). Big reading for a big future. New Reading 2, 18–19. Available at: http://www.cnki.com.cn/Article/CJFDTOTAL-XYDU202102012.htm

[ref24] Li Y. (2022). Analysis of the current situation of extracurricular reading of primary school students and strategies for improvement--an example of a primary school in Chengguan District, Lanzhou City. Gansu Education 21, 60–68.

[ref25] Li G. Sun Z. Zhen F. Ji X. R. Gunderson L. (2022). Home literacy environment and Chinese-Canadian first graders’ bilingual vocabulary profiles: a mixed methods analysis. Sustainability 14:15788. doi: 10.3390/su142315788

[ref26] Li H. Wu D. Degotardi S. Chik A. (2022). Family language policy and bilingual parenting in monolingual Beijing: latent profiles and associated predictors. Int. J. Biling. Educ. Biling. 25, 3374–3388. doi: 10.1080/13670050.2022.2058867

[ref27] Liao W. Lee T. Jiang W. Chao C. (2019). Augmented reality teaching system based on cognitive theory of multimedia learning-an example system on four-agent soup. Appl. Sci. Manag. Res. 6, 54–69. doi: 10.6511/ASMR.201909_6(1).0005

[ref28] Lipner M. Armon-Lotem S. Walters J. Altman C. (2021). Crosslinguistic influence (CLI) of lexical breadth and depth in the vocabulary of bilingual kindergarten children–a bilingual intervention study. Front. Psychol. 12:671928. doi: 10.3389/fpsyg.2021.671928, PMID: 34658996 PMC8516401

[ref29] Liu D. (2016). The language proficiency test for children. Tianjin: Tianjin University Press.

[ref30] Liu X. Wang T. Zhang G. (2019). Exploring the professional publishing path of AR picture books: Based on the comparison of parent-child interaction in reading AR and paper picture books. Sci. Technol. Publish. 11, 83–86. doi: 10.16510/j.cnki.kjycb.2019.11.025

[ref31] Lv G. Yuan Q. Wang J. (2020). Learning status of left-behind children in rural elementary school and suggestions for helping them. Rural Econ. Technol. 31, 346–349. doi: 10.3969/j.issn.1007-7103.2020.09.145

[ref32] Ma L. (2021). Research on the development process of recommended extracurricular Reading bibliography for primary and secondary school students. J. Fujian Library Sci. 2, 42–48.

[ref33] Ma J. (2022). Research on the application status and optimization strategies of information Technology in Primary School Chinese Teaching. New Curriculum 18, 156–157.

[ref34] Ma H. Qie Y. (2022). Difficulties in the education of left-behind and migrant children. J. Hengshui College 4, 106–111. doi: 10.3969/j.issn.1673-2065.2022.04.020

[ref35] Mayer R. (2009). Multimedia learning. 2nd Edn. Cambridge: Cambridge University Press.

[ref36] Mei B. Brown G. T. Teo T. (2018). Toward an understanding of preservice English as a foreign language teachers’ acceptance of computer-assisted language learning 2.0 in the People’s republic of China. J. Educ. Comput. Res. 56, 74–104. doi: 10.1177/0735633117700144

[ref37] National Language Commission. (1997). Standard of Putonghua proficiency test level. Beijing: National Language Commission.

[ref38] Pan Y. Jiang S. (2020). Research on the design of English microlearning under the perspective of multimedia learning theory. Teach. Manag. 27, 102–108.

[ref39] Patrick R. (2019). Comprehensible input and Krashen's theory. J. Classics Teach. 20, 37–44. doi: 10.1017/S2058631019000060

[ref9001] Shu X. Gu X. (2023). An Empirical Study of A Smart Education Model Enabled by the Edu-Metaverse to Enhance Better Learning Outcomes for Students. Systems, 11:75. doi: 10.3390/systems11020075

[ref40] Van den Bosch L. J. Segers E. Verhoeven L. (2020). First and second language vocabulary affect early second language reading comprehension development. J. Res. Read. 43, 290–308. doi: 10.1111/1467-9817.12304

[ref41] Vnucko G. Klimova B. (2023). Exploring the potential of digital game-based vocabulary learning: a systematic review. Systems 11:57, 2079–8954. doi: 10.3390/systems11020057

[ref42] Wang B. Luo X. Yue A. Tang L. Shi Y. (2022). Family environment in rural China and the link with early childhood development. Early Child Dev. Care 192, 617–630. doi: 10.1080/03004430.2020.1784890

[ref43] Wei X. (2019). The role of English phonological processing skills, word decoding, and general cognitive ability in Chinese children’s English literacy development. Foreign Lang. Teach. 40, 61–65+112. doi: 10.16362/j.cnki.cn61-1023/h.2019.05.012

[ref44] Wei A. (2020). The Application Research of English Picture Book in Reading Teaching in the Middle Grades of Primary School ——Taking the Yilin Version of “Following the Rabbit Graded English Picture Book” as An Example. [Master’s thesis]. Hangzhou: Hangzhou Normal University.

[ref45] Xiong T. Feng A. (2020). Localizing immersion education: a case study of an international bilingual education program in South China. Int. J. Biling. Educ. Biling. 23, 1125–1138. doi: 10.1080/13670050.2018.1435626

[ref46] Xue H. Deng R. Chen Y. Zheng W. (2022). How does bilingual experience influence novel word learning? Evidence from comparing L1-L3 and L2-L3 cognate status. Front. Psychol. 13:3199. doi: 10.3389/fpsyg.2022.1003199, PMID: 36506949 PMC9731340

[ref47] Yang J. (2021). A Group Work Study on Cultivating Extracurricular Reading Habits of Rural Left-Behind children. [Master’s thesis]. Ji'an: Jinggangshan University.

[ref48] Yao Y. (2017). Innovations and Reflections on Contemporary Primary School Picture Book Teaching in China. Curriculum. Teach. Materials Pedagogy 37, 94–99. doi: 10.19877/j.cnki.kcjcjf.2017.10.016

[ref49] Yu S. (2018). Study on the Mode of Parent-child Co-reading of Different Types of Picture Books for Children Aged 4-6. [Master’s thesis]. Shanghai: Shanghai Normal University.

[ref50] Zhang L. (2018). The Influence of Picture Book Reading Teaching on Primary School Students’ English Learning. [Master’s thesis]. Jinan: Shandong Normal University. Available at: http://cdmd.cnki.com.cn/Article/CDMD-10445-1018220889.htm

[ref52] Zhang W. (2021). The current situation of information technology application in elementary school language teaching and improvement strategies. Hundred Home of Prose 3, 173–180.

[ref53] Zhang X. Yan F. Chen Y. (2021). A floating dream: urban upgrading, population control and migrant children’s education in Beijing. Environ. Urban. 33, 11–30. doi: 10.1177/0956247820976850

[ref51] Zhang Y. (2019). Analysis of the Current Situation of Elementary School Students’ Digital Reading and Research on Countermeasures. [Master’s thesis]. Shanghai: Shandong Normal University.

[ref54] Zhou G. (2020). An analysis of mobile children’s learning behavior characteristics and optimization strategies. Exam. Weekly 9, 31–37.

